# Incidence and risk factors for surgical site infections in obstetric and gynecological surgeries from a teaching hospital in rural India

**DOI:** 10.1186/s13756-017-0223-y

**Published:** 2017-06-14

**Authors:** Ashish Pathak, Kalpana Mahadik, Manmat B. Swami, Pulak K. Roy, Megha Sharma, Vijay K. Mahadik, Cecilia Stålsby Lundborg

**Affiliations:** 1 Department of Paediatrics, Ruxmaniben Deepchand Gardi Medical College, Ujjain, Madhya Pradesh India; 2 Department of Women and Children’s Health, International Maternal and Child Health Unit, Uppsala University, Uppsala, Sweden; 3 Global Health - Health Systems and Policy, Department of Public Health Sciences, Karolinska Institutet, Stockholm, Sweden; 4 Department of Obstetrics and Gynecology, Ruxmaniben Deepchand Gardi Medical College, Ujjain, Madhya Pradesh India; 5 Department of Pharmacology, Ruxmaniben Deepchand Gardi Medical College, Ujjain, Madhya Pradesh India; 6 Ruxmaniben Deepchand Gardi Medical College, Ujjain, Madhya Pradesh India

**Keywords:** Surgical site infection, Healthcare associated infections, Obstetric and gynecological surgeries, Incidence, Risk factors, India

## Abstract

**Background:**

Surgical site infections (SSI) are one of the most common healthcare associated infections in the low-middle income countries. Data on incidence and risk factors for SSI following surgeries in general and Obstetric and Gynecological surgeries in particular are scare. This study set out to identify risk factors for SSI in patients undergoing Obstetric and Gynecological surgeries in an Indian rural hospital.

**Methods:**

Patients who underwent a surgical procedure between September 2010 to February 2013 in the 60-bedded ward of Obstetric and Gynecology department were included. Surveillance for SSI was based on the Centre for Disease Control (CDC) definition and methodology. Incidence and risk factors for SSI, including those for specific procedure, were calculated from data collected on daily ward rounds.

**Results:**

A total of 1173 patients underwent a surgical procedure during the study period. The incidence of SSI in the cohort was 7.84% (95% CI 6.30–9.38). Majority of SSI were superficial. Obstetric surgeries had a lower SSI incidence compared to gynecological surgeries (1.2% versus 10.3% respectively). The risk factors for SSI identified in the multivariate logistic regression model were age (OR 1.03), vaginal examination (OR 1.31); presence of vaginal discharge (OR 4.04); medical disease (OR 5.76); American Society of Anesthesia score greater than 3 (OR 12.8); concurrent surgical procedure (OR 3.26); each increase in hour of surgery, after the first hour, doubled the risk of SSI; inappropriate antibiotic prophylaxis increased the risk of SSI by nearly 5 times. Each day increase in stay in the hospital after the surgery increased the risk of contacting an SSI by 5%.

**Conclusions:**

Incidence and risk factors from prospective SSI surveillance can be reported simultaneously for the Obstetric and Gynecological surgeries and can be part of routine practice in resource-constrained settings. The incidence of SSI was lower for Obstetric surgeries compared to Gynecological surgeries. Multiple risk factors identified in the present study can be helpful for SSI risk stratification in low-middle income countries.

## Background

Postoperative surgical site infections (SSI) are an important health care associated (HAI) infection and one of the most frequent causes of post-operative morbidity [1]. In high-income countries, approximately 2% of surgeries are affected by SSIs [1, 2]. Although the rates of SSI are low in United States of America (USA) and European countries it is second frequent type of HAI [1]. World Health Organization (WHO) shows that SSIs are most frequently reported type of HAI in low and middle-income countries (LMICs) with a pooled incidence of 11.8 episodes of SSI per 100 surgical procedures [1].

In high-income countries the SSI rates for gynecological surgeries are similar to that of other surgical procedures [3]. Hysterectomy for gynecological causes is reported to have a SSI rate of 1.7% according to the Centre for Disease Control (CDC), USA data [3–5]. SSIs are the second most common complication after urinary tract infections as HAI in cesarean delivery (CD) with reported rates between 3 to 15% in USA [6–8] and a cumulative rate of 2.9% in European Centre for Disease Control data from 20 networks in 15 European Union countries [9]. However, the rates of SSI following CD have varied from 10 to 20% in studies from LMIC’s [1, 10–12].

In USA an episode of SSI among the gynecological surgeries, doubles the episode cost of care and triples the risk of re-admission [13]. Because of these financial implications the efforts to improve quality of surgery in high-income countries have been initiated. Some such efforts include the CDC’s National Health Safety Network (NHSN) and American College of Surgeon’s National Surgical Quality Improvement Program in USA [14, 15]. These initiatives have focused on establishing standard methods of data collection on risk factors for SSI and 30-day post discharge surveillance [14, 15]. However, the NHSN SSI risk model for obstetric surgeries does not have more than 3 independent variables for predicting SSI [14]. Also, there are limited numbers of study that simultaneously report SSI from both obstetric and gynecological surgeries [12, 16, 17]. Simultaneous reporting of SSI rates from both obstetric and gynecological surgeries would give a more complete picture of SSI. Therefore, the aim of the present study was to estimate the occurrence of 30-day postoperative SSI within a single gynecology and obstetric department and to identify the associated risk factors. To the best of our knowledge this is the first study in India reporting incidence and risk factors associated with Obstetric and Gynecological surgeries simultaneously.

## Methods

### Setting

The study was carried out at the Department of Obstetrics and Gynecology in the Chandrikaben Rashmikant Gardi Hospital (CRGH), which is situated in a rural area of Madhya Pradesh in India. The CRGH is a 600-bed teaching, tertiary care hospital within the private, not-for-profit health care sector and attached to RD Gardi Medical College (RDGMC), Ujjain.

### Patient characteristics

#### Duration of study

The study was conducted on consecutive women admitted in CRGH from September 2010 to February 2013.

#### Inclusion and exclusion criteria

The inclusion criterion was to have undergone a major Obstetrics and/or Gynecological surgery at the CRGH. Patients that stayed in the hospital for less than 24 h post-operatively were not included in the study.

#### List of obstetric and gynecological procedures performed

The obstetric operative procedures performed were: lower segment cesarean section (LSCS) for cesarean deliveries (CD), exploratory laparotomy for ectopic pregnancy and dilatation and curettage for medical termination of pregnancy. The gynecological procedures performed were: vaginal hysterectomy for 2nd or 3rd degree utero-vaginal discharge; abdominal hysterectomy for fibroids or dysfunctional uterine bleeding; radical hysterectomy for invasive carcinomas (for example endometrial carcinoma and ovarian cancer). Exploratory laparotomy for ovarian cancer; anterior colporraphy for cystocele; cervical biopsy for suspected cervical cancer; polypectomy for endometrial polyp; tubal ligation and tubectomy; dilatation and curettage for dysmenorrhea or dysfunctional uterine bleeding. No laparoscopic procedures were done during the study period.

#### Risk factors for SSI

Risk factors for SSI were explored and grouped in following categories: demographic features, peri-operative co-morbid conditions like number of vaginal examinations up-to 48 h prior to surgery, presence of vaginal discharge, elevated blood sugar (due to either Diabetes mellitus or gestational diabetes). Presence of medical disease defined as presence of any of the following: hypertension (either essential or pregnancy induced), severe anemia defined as preoperative hemoglobin <7 g%, or presence of any diagnosed systemic disease like renal, heart disease, or liver disease. Requirement of blood transfusion pre-surgery, during or 48 h post surgery was recorded. American Society of Anesthesia (ASA) class was dichotomized in ASA class 1 or class 2 (as one class) and ASA class 3 or more [18]. Intraoperative factors that were explored were: emergency or non-emergency surgery, type of anesthesia, wound classification, concomitant other surgical procedures and operative time. Operative time was measured as the time from skin incision to completion of skin closure and was classified as less than or up-to one hour, between 1 to 4 h and more than 4 h. An operative time more than 4 h was considered a risk factor for SSI based on previous work done by Culver et al. [14]. Administration of antibiotic prophylaxis with intravenous Cefazolin in the dose of 1 g given one hour prior to surgery and continued for maximum up-to 48 h was considered appropriate practice. Any deviation from above practice was noted as inadequate prophylaxis. Duration of pre and postoperative stay was calculated in days and compared for patients with and without SSI’s.

#### Surgical preparation

All procedures followed the same protocol. Shaving was done 24 h before surgery using razors. Surgical site was prepared with alcohol-povidone iodine-alcohol sequence. The initial dressing check was done 48 h after surgery.

### Provider characteristics

During the study period, the Department of Obstetrics and Gynecology had two operating units, having 30 beds each, distributed in two wards. Each unit had at least three residents and teaching faculty consisting of a professor, an associate professor, and an assistant professor. The teaching and surgical experience of each professor was a minimum of 8 years, associate professor 5 years and assistant professor at least 3 years. A senior and a junior level resident, under the supervision of at least one teaching faculty, assisted all the operative procedures.

### Data collection

A surveillance system as suggested by the CDC NHSN criteria were used for diagnosing SSI [19]. SSI was defined as a wound swab culture confirmed infection at the site of surgery within 30 days after an operation [19]. The SSIs were classified as: superficial incisional/vaginal cuff cellulitis, deep incisional or pelvic cellulitis organ/space infections as per NHSN criteria [19].

Data were collected prospectively using predefined data collection forms developed after literature review to identify risk factors for SSI and also after local expert group consultations. Trained research assistant collected information daily on all study participants after obtaining informed written consent, and followed them until discharge, completed a form containing potential risk factors for SSI, sent wound swab for culture as appropriate and completed post discharge surveillance. The post discharge surveillance was done by actual patient visit in majority of patients (81%) or by mobile phones, as described elsewhere [20]. The in-charge teaching faculty, junior and senior resident, did direct surgical site surveillance by inspecting the surgical site 48 to 72 h after surgery.

### Statistical analysis

Data were entered in EpiData Software (version 3.1, EpiData Software Association, Odense, Denmark) and transferred to Stata (version 11.0; Stata Corp, College Station, TX) for analyses. Frequencies and percentages were determined for binary and categorical variables. Range and means were calculated for continuous variables. Overall and procedure specific cumulative incidence rate of SSI was also calculated.

Pearson Chi-square test was used to test for each risk factor’s association with SSI. Stepwise multivariate logistic regression with backward elimination was used to develop the model. In bivariate analyses, a *P* value of <0.1 was considered significant for entry into the model, with SSI as outcome variable. The analysis was started with the full model, then the variable with highest *P* value was removed, and model was revised and refitted with remaining predictors. The procedure was repeated till the *P* value of all the predictor variables was less than 0.1 except age; we designated this as final model. Adjusted odds ratios and their respective 95% confidence intervals (CI) were then calculated from the final model. A *P* value of < .05 was considered significant in the final model. For the final model, model discrimination was done using C-statistics-receiver-operating-characteristics (ROC) curve, while model calibration was done using Hosmer-Lemeshaw “goodness-of-fit” test [21–23]. The ROC quantifies the ability of the model to accurately predict risk factors that will lead to development of SSI versus those that will not. A value more than 0.75 (and near one) predict excellent discrimination [21, 22]. A better model calibration is measured by a high *P*-value of the chi-square value in the Hosmer-Lemeshaw “goodness-of-fit” test [22]. Ethical permission for the study was obtained from the Ethics Committee of RDGMC (approval number 114/2010)

## Results

### Demographics

Of the 2560 admitted patients, 1173 underwent a surgical procedure. These patients constituted the final cohort. Age ranged from 10 to 80 years (mean ± standard deviation: 37.3 ± 13.6). Three patients died during the hospital stay in the study period (3/1173;0.2%). SSI was not suspected in any of the patient that died. The majority (75%) of surgeries performed were elective procedures. Most surgeries were clean (78%) or clean contaminated (18%). Very few surgeries were contaminated (2%). The duration of hospital stay ranged from 1 to 90 days (mean ± standard deviation: 13.8 ± 8.6 days). The mean length of postoperative stay for patients with SSI was longer by 3.61 days compared to patients without SSI (11.53 days vs. 7.92 days).

### Incidence of SSI

SSI was confirmed in 92 of the 1173 patients in the cohort. Thus, the cumulative incidence rate of SSI was 7.84% (95% CI 6.30–9.38). These SSI were further classified into superficial incisional primary (5.62%; *n* = 66), deep incisional primary (2.22%, *n* = 26). The SSI rates were lower for obstetric surgeries compared to gynecological surgeries; 1.23% (95% CI 0.02–2.4) versus 10.37% (95% CI 8.32–12.43), respectively. The list of surgical procedures performed and the distribution of the SSI rates according to the different surgical procedures is shown in Table 1. The characteristics of patients with and without SSI are given in Table 2. Procedures like anterior colporrhaphy, vaginal and radical hysterectomies were associated with higher SSI rates (between 9 to13%) whereas lower SSI rates (between 3 to 7.6%) were observed for dilatation and curettage and LSCS. The indications of LSCS in the study are shown in Table 2.

**Table 1:** Distribution of SSI rates according to surgical procedures in the cohort of 1173 patients

Surgical procedure	Number (%)	Mean SSI rate
Lower Segment Cesarean Section	239(20)	3.76
Vaginal hysterectomy	325(28)	11.6
Abdominal hysterectomy	237(20)	3.79
Radical hysterectomy	31(3)	9.03
Exploratory laparotomy	24(2)	4.16
Polypectomy	93(8)	3.22
Anterior colporrhaphy	8(1)	12.5
Dilatation and curettage	119(10)	0.84
Tubal ligation	18(1.5)	-
Cervical biopsy	19(1.5)	5.26
Others	60(5)	1.66

**Table 2:** Indications of Lower Segment Cesarean Section among the Obstetric patients in the present study

Indication	*n* = 239 (%)
Previous Cesarean delivery	74(31)
Fetal distress	38(16)
Prolonged labor	34(14)
Mal-presentation	22(9)
Obstructed labor	19(8)
Hypertensive disorder of pregnancy	22(9)
Antepartum hemorrhage	7(3)
Preterm rupture of membranes	5(2)
Others^a^	4(2)
Combination of indications	14(6)

^a^Include maternal distress, maternal request, cephalo-pelvic disproportion (big baby), twins with malpresentation, ruptured uterus, cord accidents, and failed induction of labor.

### Risk factors associated with SSI

There were no statistically significant differences between patients having superficial versus patients having deep SSI, therefore the risk factor for both are presented together.

- Bivariate analysis. Patients older than 40 years of age were more likely to have an SSI than those between 25 and 40 year of age (OR 2.95; vs. 2.19); as compared to those less than 25 years of age. The odds of SSI were almost 3 times higher for grand-multipara (5 or more pregnancies) compared to primiparous women. Number of vaginal examinations in 48 h prior to surgery, were significantly related to SSI, with odds increasing with increasing number of examinations (OR 10.00 vs. 37.67 for 1–9 examinations and >10 examinations, respectively compared with no vaginal examinations). Similarly, presence of vaginal discharge before the surgery increased the risk of SSI. Presence of high blood sugars (either Diabetes Mellitus or gestational diabetes) and medical illness (severe anemia, heart disease, hypertension and renal disease) emergency surgical procedure and use of general anesthesia during the surgery increased the risk of SSI (Table 3). ASA class 3 or higher had greater risk for SSI compared with class 1 or 2 (OR 20.28). Duration of surgery of more than 4 h increased risk of SSI (OR 5.67), and also incorrect timing of antibiotic prophylaxis was associated with increased risk of SSI (Table 3).
- Multivariate analysis. The results of final fitted model (Table 4) showed that with increase in the age the risk of SSI increased (OR 1.03, 95% CI 1.01–1.06; *P* = 0.001). Compared to no vaginal examination, each vaginal examination increased the risk of SSI by 31% (OR 1.31, 95% CI 1.23–1.40; *P* < 0.001). Presence of vaginal discharge at the time of admission also increased the risk of SSI (OR 4.04, 95% CI 1.77–9.20; *P* = 0.001). Presence of a medical disease increased the risk of SSI by nearly 6 folds (OR 5.76, 95% CI 2.84–11.67; *P* < 0.001). An ASA class of greater than 3 was associated with increased risk of SSI (OR 12.8, 95% CI 5.32–30.79; *P* < 0.001). A concomitant surgery increased the risk of SSI (OR 3.26, 95% CI 1.36–7.83; *P* = 0.008). Each increase in hour of surgery, after the first hour, doubled the risk of SSI (OR 2.02, 95% CI 1.53–2.66; *P* < 0.001). If the antibiotic prophylaxis timing was inappropriate i.e. not within one hour of surgery, it increased the risk of SSI by nearly 5 times (OR 4.91, 95% CI 2.04–11.81; *P* < 0.005). Each day increase in stay in the hospital after the surgery increased the risk of contacting an SSI by 5% (OR 1.058, 95% CI 1.01 to 1.10; *P* = 0.007).
- Model performance. The ROC of the final model was 0.9513 showing excellent model fit. The Hosmer-Lemeshaw test showed that chi-square was 5.69 (*P* = 0.6823) showing good model calibration.
- Power calculation. With 1173 patients that underwent surgery in the cohort we could detect a change in SSI rate (dependent variable) in the cohort from 3 to 10% with at least 10% variation in predictor (independent variables) with a power of 99%.

**Table 3:** Univariate analyses of demographic, preoperative, and intraoperative characteristics of patients with and without surgical site infection in the cohort of 1173 patients

Variable	Total (*n* = 1173)	No SSI (*n* = 1081) (%)	SSI (*n* = 92) (%)	Odds Ratio (OR)	95% CI of OR	*P* value
Age (in years)						
10–25	310	298 (96)	12 (4)	R	-	-
> 25–40	468	430 (92)	38 (8)	2.19	1.12–4.26	0.021
> 40	395	353 (89)	42 (11)	2.95	1.52–5.71	0.001
Parity						
Primiparous	157	155 (99)	2 (1)	R	-	-
Multiparous	192	185 (96)	7 (6)	2.93	0.60–14.3	0.184
Grand-multiparous	28	27 (96)	1 (6)	2.87	0.25–32.7	0.396
Non pregnant	796	714 (90)	82 (10)	8.90	2.16–36.58	0.002
Number of vaginal examinations 48 h. before surgery						
None	890	872 (98)	18 (2)	R	-	-
1–9	187	115 (83)	32 (17)	10.00	5.47–18.26	<0.005
> 10	96	54 (56)	42 (44)	37.67	20.33–69.81	<0.005
Vaginal discharge						
Absent	1084	1022 (94)	62 (6)	R		
Present	89	59 (66)	30 (31)	5.46	3.35–8.88	<0.005
Variable	All (*N* = 1173)	No SSI (*n* = 1081) (%)	SSI (*n* = 92) (%)	Odds Ratio (OR)	95% CI of OR	*P* value
Blood glucose levels						
Normal	677	628 (93)	49 (7)	R	-	
High	92	57 (62)	35 (38)	7.86	4.7–13.12	<0.005
Medical illness^#^						
Absent	947	917 (97)	30 (3)	R	-	
Present	226	164 (73)	62 (27)	11.55	7.24–18.42	<0.005
ASA class						
Class 1 or 2	1111	1052 (95)	59 (5)	R	-	
Class 3–5	62	29 (47)	33 (53)	20.28	11.54–35.64	<0.005
Emergency surgery						
No	882	830 (94)	52 (6)	R	-	
Yes	291	251 (86)	40 (14)	2.54	1.64–3.93	<0.005
Type of anesthesia						
Local or spinal	889	839 (94)	50 (6)	R	-	
General anesthesia	284	284 (85)	42 (15)	2.91	1.88–4.49	<0.005
Concurrent surgical procedure						
No	1067	1005 (94)	62 (6)	R	-	
Yes	106	76 (72)	30 (28)	6.39	3.90–10.48	>0.005
Duration of surgery (hr.)						
Less than 1	262	250 (95)	12 (5)	R	-	-
1–4	852	787 (92)	65 (8)	1.72	0.91–3.23	0.92
More than 4	41	26 (63)	15 (37)	5.67	5.08–28.39	<0.005
Antibiotic prophylaxis						
Within 1 h	524*	512 (98)	12 (2)	R	-	
After 1 h	640*	562 (88)	78 (12)	5.92	3.18–11.00	<0.005
Blood transfusion						
No	1033	969 (94)	64 (6)	R	-	
Yes	140	112 (73)	28 (20)	3.78	2.32–6.14	<0.005
Duration of post-op stay						
1–7 days	663	634 (96)	29 (4)	R	-	-
> 7–14 days	424	383 (90)	41 (10)	2.34	1.43–3.82	0.001
> 14 days	86	64 (74)	22 (26)	7.51	4.07–13.84	<0.005

**Table 4:** Multivariate analyses of demographic, preoperative, and intraoperative characteristics of patients with and without surgical site infection in the cohort of 1173 patients

Variable	Adjusted odds ratio (OR)	95% CI of OR	*P* value
Age (Continuous)	1.03	1.01–1.06	0.005
Number of vaginal examinations (Continuous)	1.31	1.23–1.40	<0.001
Vaginal discharge	4.04	1.77–9.20	0.001
Presence of medical illness	5.76	2.84–11.67	<0.001
ASA class >3	12.80	5.32–30.79	<0.001
Concomitant surgery	3.26	1.36–7.83	0.008
Surgical time (Continuous)	2.02	1.53–2.66	<0.001
Inadequate antibiotic prophylaxis	4.91	2.04–11.81	<0.005
Length of stay (Continuous)	1.058	1.01–1.10	0.007

## Discussion

To the best of our knowledge this is the first study in India reporting incidence and risk factors associated with Obstetric and Gynecological surgeries simultaneously. A study from Tanzania reported a SSI rate of 10.9% among 774 patients undergoing CD [11]. A study from Estonia reported a SSI rate of 6.2% among 305 patients that underwent cesarean section deliveries and had a 30-day follow-up post surgery [24]. A cohort of women with CD from Thai-Myanmar border showed a SSI rate of 5.9% [25]. All the above studies have reported a higher rate of SSI among the CDs as compared to our study. An Italian study reported an SSI rate of 4.7% from 430 mothers with CD included in the study [26]. A study from Israel reported an SSI rate of 3.7%, which is similar to our study [27]. One reason for low rate of SSI in our study could be due to high proportion of patients belonging to clean or clean contaminated wounds. It is well known that patients with contaminated wounds have nearly three-fold increased risk of SSI compared to non-contaminated wounds [1, 14]. Other reason could be due to the fact that most of the CDs were done within an hour. This might be due to experience of the operating team. However, it has been shown that CDs performed by resident teaching services increased the risk for SSI (OR 2.15) [8]. This might be due to lack of surgical experience among the operating residents.

Most of the SSIs found in our study were superficial SSI. This is similar to other studies from resource-constrained settings [11, 25]. In USA also approximately two-thirds of the SSI are superficial and remaining deep [3].

Gynacological surgeries showed higher SSI rates compared to obstetric procedures (Table 1). In USA the estimated incidence of SSIs in hysterectomy is around 1.7% [3]. However, according to the authors this seems to be an underestimate as many hospitals lack the resources to track SSI occurring outside of the hospital (up-to 30 days postoperatively) [3].

Young maternal age has been shown to be a risk factor for SSI following cesarean section. However, our study we found that age more than 40 years as a risk factor for SSI. This may be due to the patient mix in our study, which included both gynecological and obstetric surgeries. A regional collaborative data from USA did not show any difference in age of the women having SSI following hysterectomy [28]

Abdominal route of hysterectomy has been shown to have a higher risk for SSI when compared to vaginal hysterectomy.

Longer operative time by more than 30 min increases the risk for SSI (OR 1.30) [28]. Another study identified that an operative time longer than 75th percentile increased the risk of SSI by 1.84 times compared to vaginal approach [4]. Duration of surgery longer than one hour has been shown to be associated with increased risk for SSI. A longer operative time is probably a proxy for complicated surgery [7]. Other explanations for association between longer surgical time and risk for SSI include: inadequate dosing of prophylactic antibiotic, tissue trauma due to instrumentation and manipulation, increased risk for hypoglycemia and hypothermia, increased blood loss, exposure to environmental pathogens, and risk for breech of sterile technique [7, 29, 30].

Inappropriate timing of antibiotic prophylaxis in our study was a significant risk factor for SSI. Similar results have been reported earlier and are also in line with recommendations from prophylaxis guidelines [31]. However, during the study period no standard policy for prophylaxis was followed. The American Congress of Obstetricians and Gynecologists recommends pre-operative antibiotic prophylaxis for hysterectomies, induced abortions, hysterosalpingography, and uro-gynacological procedures [31].

Multiple vaginal examinations and presence of vaginal discharge increased the risk for SSI in our study. This may be due to ascending infection leading to secondary spread to the surgical site. Another study reported that multiple vaginal examinations (more than three) increased the risk for SSI by 2.6 times [11]. Another study identified chorioamnionitis as a major risk factor for SSI post cesarean delivery [8].

We grouped presence of medical diseases post-hoc because of small number of patients with individual medical disease. Risk factors including medical diseases identified for SSI from two large studies are: obesity, diabetes, smoking, respiratory infection, poor nutrition, ASA score greater than 2, and low socio-economic status [4, 7]. An ASA score of more than three was reported to increase the risk of SSI by 1.52 times [28]. A study on SSI following cesarean section from Tanzania showed that ASA score more than 3 had about 2.7 times higher risk for SSI [11]. For superficial SSI the risk was shown about 1.52 times for gynecological surgeries [28], while another study showed the risk as 1.79 times [4]. For cesarean deliveries, a study reported the risk 1.61 times [8]. Diabetes has been significantly associated with SSI secondary to gynecological surgeries [3]. The specific risk reported in hysterectomies is 1.54 times [4]. Patients of diabetes especially with poor glycemic control share much comorbidity, like obesity, poor nutritional status, poor peripheral oxygen supply, and metabolic derangements [3]. Obesity is an uncertain risk factor for SSI in gynecological surgeries [4]. It has been shown in one study that rather than obesity, subcutaneous thickness as measure by MRI could be more important risk factor for SSI [32]. Patients with hypertensive disorder of pregnancy have been show to have 2.9 times higher risk for SSI [3]. A history of cerebrovascular accidents with neurological deficit has been shown to increase the risk of SSI by 4.41 times (95% CI 1.54–12.65; *P* < 0.001) [4]. Our study did not find an association between preoperative blood transfusion and SSI. The findings are similar to a large secondary database analysis from USA [4] and also from a large regional collaborative from Michigan [28]. However, other studies have identified association between blood transfusion and SSI [5]. An explanation for risk for SSI following blood transfusion remains unclear and probably reflects a proxy for severe anemia and consequent low oxygen carrying capacity and delivery to the tissues, potential contamination, and transfusion related immunomodulation in presence of critical illness [7, 33].

Most important strengths of our study are: 1) our study was done using standard data collection methods 2) we collected data prospectively and did not use computer generated codes 3) our risk factor analysis included a large number of variable on patient characteristics, operative detail and timing of antibiotic prophylaxis 4) we calculated procedure specific SSI rates 5) all the surgeons participated 6) due to the study design we could develop risk predicting statistical model. Our study has certain limitations. We did not collect socio-economic data and data on obesity, but most of our patients belong to low socio-economic class and are not obese, but could have malnutrition. We did not calculate the cumulative experience of the operating team for a given surgery, although we did know the overall experience of each member. This would have increased the burden on our data collection team. Finally, lack of antibiotic policy might have affected the SSI rates.

## Conclusion

Incidence and risk factors from prospective SSI surveillance can be reported simultaneously for the Obstetric and Gynecological surgeries and can be part of routine practice in resource-constrained settings. The incidence of SSI was lower for Obstetric surgeries compared to Gynecological surgeries. The study identified multiple risk factors for SSI for Obstetric and Gynecological surgeries in a tertiary rural hospital in India. Some of the risk factors identified are amendable through interventions. Thus, the multiple risk factors identified in the present study can be helpful for SSI risk stratification and prioritizing interventions in low-middle income countries.
